# Noncoding RNAs in subchondral bone osteoclast function and their therapeutic potential for osteoarthritis

**DOI:** 10.1186/s13075-020-02374-x

**Published:** 2020-11-25

**Authors:** Li Duan, Yujie Liang, Xiao Xu, Jifeng Wang, Xingfu Li, Deshun Sun, Zhiqin Deng, Wencui Li, Daping Wang

**Affiliations:** 1 Department of Orthopaedics, Shenzhen Intelligent Orthopaedics and Biomedical Innovation Platform, Guangdong Artificial Intelligence Biomedical Innovation Platform, Shenzhen Second People’s Hospital, The First Affiliated Hospital of Shenzhen University Health Science Center, Shenzhen, 518035 China; 2 Department of Child and Adolescent Psychiatry, Shenzhen Kangning Hospital, Shenzhen Mental Health Center, Shenzhen Key Laboratory for Psychological Healthcare & Shenzhen Institute of Mental Health, Shenzhen, 518003 China; 3 Hand and Foot Surgery Department, Shenzhen Second People’s Hospital, Shenzhen, 518035 Guangdong China; 4 Department of Biomedical Engineering, Southern University of Science and Technology, Shenzhen, 518055 China

**Keywords:** Noncoding RNAs, Osteoclasts, Subchondral bone remodeling, Osteoarthritis

## Abstract

Osteoclasts are the only cells that perform bone resorption. Noncoding RNAs (ncRNAs) are crucial epigenetic regulators of osteoclast biological behaviors ranging from osteoclast differentiation to bone resorption. The main ncRNAs, including miRNAs, circRNAs, and lncRNAs, compose an intricate network that influences gene transcription processes related to osteoclast biological activity. Accumulating evidence suggests that abnormal osteoclast activity leads to the disturbance of subchondral bone remodeling, thus initiating osteoarthritis (OA), a prevalent joint disease characterized mainly by cartilage degradation and subchondral bone remodeling imbalance. In this review, we delineate three types of ncRNAs and discuss their related complex molecular signaling pathways associated with osteoclast function during bone resorption. We specifically focused on the involvement of noncoding RNAs in subchondral bone remodeling, which participate in the degradation of the osteochondral unit during OA progression. We also discussed exosomes as ncRNA carriers during the bone remodeling process. A better understanding of the roles of ncRNAs in osteoclast biological behaviors will contribute to the treatment of bone resorption-related skeletal diseases such as OA.

## Introduction

Osteoclasts, originating from the mononuclear cell fusion of the monocyte/macrophage lineage, are the only cells responsible for bone resorption [1]. During processes ranging from osteoclast differentiation to bone resorption, noncoding RNAs (ncRNAs) function as epigenetic regulators. The main types of ncRNAs, including microRNAs (miRNAs), long ncRNAs (lncRNAs), and circular RNAs (circRNAs), establish an intricate network to regulate the transcription of genes involved in osteoclast function during bone resorption [2]. Recently, exosomal ncRNAs and exosomal delivery of miRNAs have attracted substantial attention during the bone remodeling process [3].

Subchondral bone is one of the basic units that constitutes the structure and function of joints, maintaining the normal structure and function of cartilage. The main characteristics of osteoarthritis (OA) are cartilage degeneration and abnormal subchondral bone remodeling. In recent years, increasing evidence has proven that osteoclast initiates subchondral bone remodeling. Aberrant subchondral bone remodeling initiates OA due to abnormal osteoclast activity [4]. However, the exact mechanisms of subchondral bone remodeling in OA progression are not fully understood. Currently, there is no ideal strategy to counteract aberrant subchondral bone remodeling in OA. A deep understanding of the roles of osteoclasts in subchondral bone remodeling will contribute to the discovery of possible molecular targets and the development of novel strategies for OA management.

In this review, we summarize ncRNAs that can maintain bone remodeling, emphasizing osteoclast function in subchondral bone resorption. We also reveal exosomes as ncRNA carriers to regulate subchondral bone osteoclasts. We endeavor to cover these topics in the context of the maintenance of subchondral bone remodeling balance in the hope of supporting the development of ncRNA-based therapeutic strategies for controlling subchondral bone osteoclasts in OA.

### Osteoclast activity in subchondral bone remodeling

Concerning the cytokines involved in subchondral bone remodeling, the RANKL/receptor activator of nuclear factor kappa-B (RANK)/osteoprotegerin (OPG) axis is a well-known critical inflammatory signaling system. RANKL induces the differentiation of monocyte-/macrophage-lineage cells into osteoclasts. OPG, a soluble secreted protein and decoy receptor, inhibits RANKL/RANK signaling. A series of studies have demonstrated that chondrocytes, osteoblasts, and osteocytes secrete RANKL and OPG, while osteoclasts express RANK. The OPG/RANKL ratio depends on the inflammation severity and is also an indication of osteoclast activity and bone metabolism under both pathological and normal physiological conditions. For instance, chondrocytes isolated from OA patients show significantly lower OPG/RANKL ratios but higher RANK/RANKL ratios than chondrocytes isolated from healthy individuals [5]. The RANKL/RANK interaction activates a series of downstream signaling pathways, including the nuclear actor kappa light chain enhancer of activated B cells (NF-κB) pathway, which stimulates c-Fos. Then, c-Fos activates nuclear factor of activated T cell cytoplasmic 1 (NFATc1), the master regulator of early-phase osteoclastogenesis. An NFATc1 autoamplification loop induces the expression of many late-phase genes, such as tartrate-resistant acid phosphatase (TRAP), cathepsin K (CTSK), and dendritic cell-specific transmembrane protein (DC-STAMP), via amplification of transcription factor (c-Fos, NF-κB, and NFATc2) activity. In addition to the action of the well-known RANKL/OPG/RANK regulatory system, the binding of colony-stimulating factor 1 receptor (CSF1R) by the growth factor macrophage colony-stimulating factor (M-CSF), which is secreted by osteoblasts, is essential for osteoclast precursor proliferation. Alternative potential osteoclastogenic molecules involved in OA development include cytokines such as interleukin (IL)-1, IL-6, IL-23, IL-17, and IL-34 [6].

Cartilage RANKL recruits osteoclasts to the subchondral plate, releasing matrix metalloproteinases (MMPs) and CTSK and thus contributing to the degeneration of focal subchondral bone and hyaline cartilage and the formation of calcified cartilage microcracks [7]. Microcrack formation and calcified cartilage degeneration further facilitate the diffusion of cartilage RANKL into subchondral bone marrow cells and subsequent recruitment of osteoclasts [7]. Massive osteoclast recruitment accelerates the degradation of the subchondral bone.

### Imbalance of subchondral bone remodeling in OA

Cartilage and subchondral bone constitute a functional bone-cartilage unit that bears weight and coordinates articular movement. Subchondral bone includes the subchondral bone plate and the underlying trabecular bone. Most previous laboratory and clinical OA studies have focused on the maintenance of cartilage integrity; less attention has been given to subchondral bone function, since the conventional treatment for early OA of the knee targets the articular cartilage [8–10]. New pathophysiological concepts have shifted attention towards the imbalance of subchondral bone remodeling as an initiator of OA-related cartilage degradation [11, 12].

Some researchers have demonstrated that changes in subchondral bone might occur at the same time as (and possibly precede) alterations in articular cartilage [13–15]. Disturbance of subchondral bone remodeling adversely affects the cartilage biomechanical environment, resulting in subsequent changes in cartilage structure and integrity and degeneration of the cartilage matrix. Thus, maintaining the balance of subchondral bone remodeling could be crucial for the treatment of early OA. Evidence obtained using physical stimuli supports this concept, e.g., extracorporeal shockwave therapy protects subchondral bone instead of articular cartilage and slows OA progression [16]. Therefore, modification of aberrant subchondral bone remodeling could be beneficial for early OA treatment.

Notably, compared with cells from healthy individuals, monocytes from OA patients display an increased capacity to differentiate into osteoclasts, suggesting that osteoclast activity is enhanced in OA patients [17]. Suppression of abnormal osteoclast activity via knee loading can prevent OA cartilage degradation and regulate subchondral bone remodeling [15]. In addition, inhibition of osteoclast activity with alendronate not only attenuates aberrant subchondral bone remodeling but also reduces innervation and pain behavior at the early stage of OA [4]. As knee pain is not only a consequence of structural deterioration in OA but also a contributor to structural decline [18], osteoclasts in subchondral bone could be promising targets for OA treatment from the standpoints of both joint structural maintenance and clinical pain management. Recently, the roles of ncRNAs in osteoclast differentiation and bone resorption have attracted much attention. A better understanding of the attraction of osteoclasts in OA and their migration to the subchondral plate may help identify other drug targets to inhibit excessive bone resorption during OA and maintain bone integrity structure and function.

### ncRNAs in osteoclasts during bone resorption

ncRNAs comprise a variety of RNA species that do not encode proteins but profoundly regulate gene transcription and translation in multiple cellular processes [19]. Several known types of ncRNAs include miRNAs, short interfering RNAs (siRNAs), piwi-interacting RNAs (piRNAs), lncRNAs, and circRNAs. Here, we focus on the involvement of miRNAs, lncRNAs, and circRNAs, as well as the intricate network of ncRNAs and ncRNA signaling pathways, in regulating osteoclast biological activity, a primary mediator of OA subchondral bone remodeling.

#### Regulation of miRNAs in osteoclasts

**Fig. 1:**
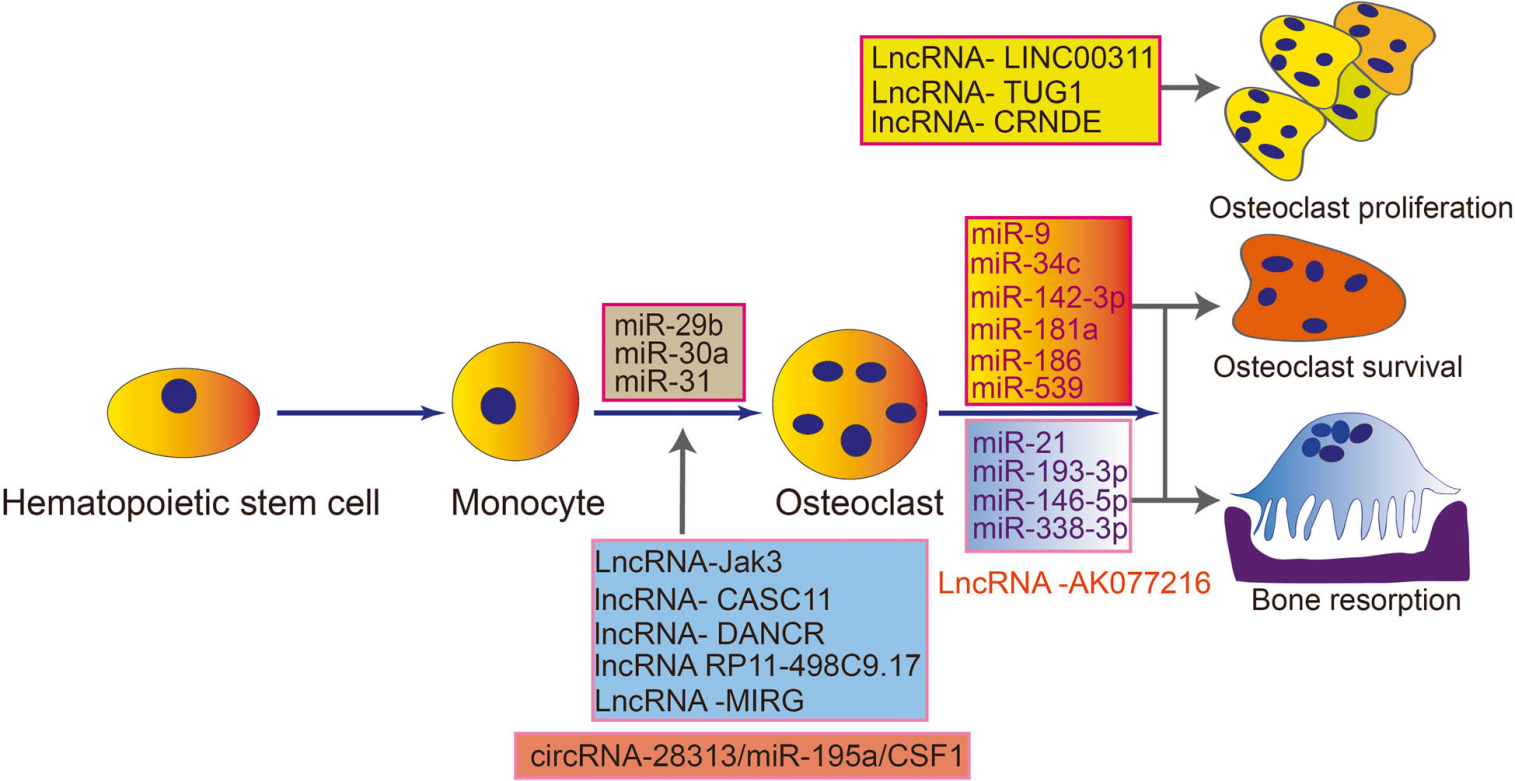
Illustration of the roles of miRNAs, lncRNAs and circRNAs as key regulators in three phases of osteoclastogenesis, osteoclast survival, and bone resorption

miRNAs are 20- to 23-nucleotide-long single-stranded ncRNA molecules that act as transcriptional repressors by binding to the untranslated regions (UTRs) of target messenger RNA (mRNA) molecules [20]. The primary function of miRNAs is to downregulate target gene expression. miRNAs are the most extensively studied ncRNAs with regard to the roles of osteoclasts in subchondral bone homeostasis and OA development (Fig. 1). As the functions of miRNAs in the commitment of progenitors to an osteoclast fate and in the late phase of osteoclast generation have been extensively reviewed [21], we focus on the functions of miRNAs in osteoclast bone resorption and survival.

#### miRNA-mediated regulation of osteoclast function and survival

Once formed, mature osteoclasts attach to the bone surface by creating a tight sealing zone over the bone surface, demarcated by a so-called actin ring, that forms around the periphery of the cell. Osteoclasts then resorb bone by secreting hydrochloric acid and proteolytic enzymes into the space underneath the sealing zone through a specialized membrane called the ruffled border. Once the process of bone resorption is complete, osteoclasts undergo apoptosis. Several studies have addressed the impacts of miRNAs on osteoclast bone resorption and fate determination [22]. In Table 1 [23–38], we have summarized the miRNAs involved in actin ring formation and apoptosis.

**Table 1:** miRNAs in osteoclast function and survival

miRNAs	Species	Up/down	Overall impact	Targets	Steps impacted	References
miR-31	Mouse	Up	Positive	RhoA	Acting ring formation	[23]
miR-21	Mouse	Up	Positive	PTEN	Resorption	[24]
miR-146-5p	Human	Up	Positive	ND	Resorption	[25]
miR-34c	Mouse	Up	Positive	LGR4	Survival	[26]
miR-29b	Mouse	Up	Positive	Bmf	Survival	[27]
miR-539	Rat	Down	Positive	ND	Survival	[28]
miR-21	Mouse	Up	Negative	FASL	Survival	[29]
miR-21	Human	Up	Negative	OPG	Resorption	[30]
miR-26a	Mouse	ND	Negative	ND	Resorption	[31]
miR-29b	Human	Down	Negative	FOS; MMP-2	Acting ring formation	[32]
miR-30a	Mouse	Down	Negative	DC-STAMP	Acting ring formation	[33]
miR-9; miR-181a	Mouse	ND	Negative	Cbl	Survival, mobility	[34]
miR-142-3p	Human	Down	Negative	PRKCA	Survival	[35]
miR-186	Mouse	ND	Negative	CTSK	Survival	[36]
miR-193-3p	Mouse	ND	Negative	NFATc1	Resorption	[37]
miR-338-3p	Mouse	Down	Negative	IKKβ	Resorption	[38]

#### miRNAs that facilitate osteoclast function and survival

Among the studied miRNAs, miR-31 is one of the most upregulated miRNAs during osteoclastogenesis in mouse bone marrow-derived monocytes/macrophages (BMMs). Neutralization of miR-31 significantly disrupts actin ring formation and thus impairs bone resorption. MiR-31 knockout and luciferase activity assays have demonstrated that Ras homologous A (RhoA) is the putative target of miR-31. Both inhibition and excessive activation of RhoA have been reported to inhibit actin ring formation and bone formation [23]. These results suggest that a moderate level of RhoA activity is crucial for the maintenance of the actin sealing ring.

Other relevant miRNAs that enhance osteoclastic bone resorption ability include miR-21 and miR-146-5p. miR-21 promotes osteoclastogenesis and bone resorption by activating the phosphatidylinositol 3′-kinase (PI3K)-Akt signaling pathway by targeting phosphatase and tensin homolog (PTEN) [24]. The PI3K-Akt signaling pathway promotes osteoclast differentiation and activation, as well as cell survival. PTEN may reverse the action of PI3K-Akt. Although miR-146-5p has been shown to play a role in enhancing bone resorption [25], its targets await further investigation.

Some miRNAs have been shown to promote osteoclast survival by inhibiting osteoclast apoptosis, such as miR-34c [26], miR-29b [27], and miR-539 [28]. miR-34c promotes osteoclast survival by targeting leucine-rich repeat-containing G-protein-coupled receptor 4 (LGR4). LGR4 can bind RANKL to activate glycogen synthase kinase-3β (GSK3β) (an NF-κB inhibitor) and lead to osteoclast apoptosis. Another relevant miRNA involved in osteoclast fate determination is miR-29b, which enhances osteoclast survival by targeting the proapoptotic factor BCL-2-modifying factor (Bmf) [27].

#### miRNAs that inhibit osteoclast function and survival

Several miRNAs [30–32] have been shown to play inhibitory roles in osteoclastic bone resorption and to form an intricate network that prevents excessive resorption activity. For example, osteoclast actin ring formation and bone resorption ability are enhanced when the expression level of miR-30a is downregulated in osteoclast precursor cells. miR-30a attenuates osteoclastogenesis via suppression of the DC-STAMP-c-Fos-NFATc1 signaling pathway [33], which is the master transcription axis of osteoclast differentiation. In addition, miR-29b [32] inhibits osteoclastogenesis via downregulation of NAFTc-1.

Other miRNAs that inhibit osteoclastic bone resorption ability include miR-21 [30], miR-26a [31], miR-193-3p [37], and miR-338-3p [38]. As mentioned above, miR-21 promotes osteoclastogenesis and bone resorption by targeting PTEN but can also negatively regulate bone resorption by increasing OPG, which contributes to maintaining bone resorption at a moderate level.

Concerning the negative roles of miRNAs in osteoclast survival, miR-21 [29], miR-9 [34], miR-142-3p [33], and miR-186 [36] have been shown to inhibit osteoclast survival by enhancing osteoclast apoptosis. In the physiological state, these miRNAs coincide with the miRNAs that facilitate osteoclast function and survival, thus modulating osteoclast behavior and bone remodeling. However, inflammation could inhibit the negative role of these miRNAs, tipping to osteoclastic bone resorption.

#### LncRNA-mediated regulation in osteoclasts

LncRNA function range from controlling nuclear architecture and transcription in the nucleus to modulating mRNA stability, translation, and post-translational modifications in the cytoplasm. LncRNAs have been identified to regulate many essential biological processes, including osteoclast-related processes (Table 2) [39–47]. They have also been found to be abnormally expressed in a growing number of diseases, including during the development of OA.

**Table 2:** LncRNAs involved in osteoclast generation

lncRNAs	Species	Up/down	Overall impact	Targets	Steps impacted	References
LncRNA-Jak3	RAW264.7	Up	Positive	NFATc1	Osteoclast differentiation	[39]
LncRNA-LINC00311	Rat osteoclasts	ND	Positive	DLL3	Induces proliferation and inhibits apoptosis of osteoclasts	[40]
LncRNA-AK131850	Mouse osteoclasts	Up	Positive	miR-93-5p	Promotes vasculogenesis of endothelial progenitor cells	[41]
LncRNA-TUG1	BMMs	Up	Positive	ND	Promotes the proliferation and inhibits the apoptosis of osteoclasts	[42]
LncRNA-AK077216	RAW264.7; BMMs	Up	Positive	NFATc1	Promotes osteoclast differentiation and enhances osteoclast bone resorption	[43]
LncRNA-CRNDE	Human osteoclasts	Up	Positive	ND	Promotes osteoclast proliferation	[44]
LncRNA- DANCR	hOCPs; RAW264.7	Up	Positive	FOXO1	Promotes osteoclastic differentiation	[45]
LncRNA RP11-498C9.17	PBMs	Down	ND	ND	Controls osteoclastogenesis.	[46]
LncRNA -MIRG	RAW264.7	Up	Positive	miR-1897	Promotes osteoclastic differentiation	[47]

To date, there have been two studies about lncRNA expression profiles during different stages of osteoclastogenesis. In one study, a lncRNA microarray expression data set was generated from RAW264.7 cells that were stimulated with RANKL to undergo osteoclastogenesis. The study identified 4348 lncRNAs that were differentially expressed in preosteoclasts; 1643 lncRNAs were upregulated, and 2705 were downregulated. In mature activated osteoclasts, 1896 lncRNAs were upregulated, and 2706 lncRNAs were downregulated. Through Gene Ontology and Kyoto Encyclopedia of Genes and Genomes biological pathway analyses, Dou et al. investigated the potential functions of the differentially expressed lncRNAs. The authors also found two downregulated lncRNAs, Gm12310 and Gm12308, that were associated with the tumor necrosis factor ligand superfamily member (Tnfsf) 12 and Tnfsf13 protein-coding transcripts, which have previously been implicated in osteoclastogenesis. However, no functional characterization was performed to test the requirements for these lncRNAs in osteoclastogenesis [2].

Another study addressing the functions of lncRNAs in osteoclastogenesis evaluated the differences in the profiles of BMMs isolated from C57/BL6 mice at two different stages of osteoclast differentiation/maturation, namely, preosteoclasts and mature osteoclasts. Of the lncRNAs that were differentially expressed, 471 were upregulated and 366 were downregulated in preosteoclasts, 639 were upregulated-regulated, and 975 were downregulated-regulated in mature osteoclasts. Further analysis revealed that 382 lncRNAs were upregulated and 259 lncRNAs were downregulated at both early and later stages of osteoclastogenesis. These results show that the lncRNA expression profile is highly regulated during osteoclastogenesis. This study also found that upregulation of lncRNA-NONMMUT037835.2 inhibited osteoclast differentiation, whereas downregulation of lncRNA-NONMMUT037835.2 promoted osteoclast formation and fusion. LncRNA-NONMMUT037835.2 inhibited osteoclastogenesis by negatively regulating RANK expression and inhibiting the NF-κB/mitogen-activated protein kinase (MAPK) signaling pathway [48]. Given the different origins of osteoclast precursors and the limited sample sizes in this study, future studies with larger sample sizes should be performed to obtain more comprehensive lncRNA expression profiles.

The involvement of lncRNA-DANCR in osteoclastogenesis and bone resorption has been investigated both in vitro and in vivo [45]. In that study, this lncRNA was found to be significantly upregulated during osteoclastogenesis and to be highly expressed in the periodontal ligament (PDL) tissues of a rat orthodontic tooth movement model. In vitro, knockdown of DANCR in PDL cells inhibited increases in Jagged1, RANKL, and IL-6 levels in the supernatants of cells treated with compression force, while overexpression of Jagged1 changed the effects of DANCR-specific siRNA (si-DANCR). In addition, human osteoclast precursor cells were cultured with culture medium from PDL cells in different treatment groups, and knockdown of DANCR significantly reduced the numbers of osteoclasts. Further RNA immunoprecipitation (RIP) and RNA pull-down assays verified that DANCR binds to Jagged1. Jagged1 is a ligand of Notch and a critical activator in the Notch signaling pathway. The Notch pathway is an evolutionarily conserved cell-cell signaling pathway that has previously been implicated in the normal development of various organs. It also contributes to pathological mechanisms, such as orthodontically induced inflammatory root resorption [49]. Thus, DANCR could positively regulate osteoclastogenesis by targeting Jagged1. On the other hand, DANCR negatively regulates osteoblast differentiation by modulating the expression of Forkhead Box O1 (FOXO1) [45], a positive regulator of bone formation. In osteoclast precursors, FOXO1 is a transcription factor that mediates the effects of RANKL on NFATc1 and several downstream effectors (DC-STAMP, ATPase H^+^ transporting V0 subunit D2 (ATP6V0D2), CTSK, and integrin ανL) during osteoclast differentiation and bone resorption [50].

#### circRNA-mediated regulation in osteoclasts

circRNAs are a novel type of endogenous single-stranded ncRNA and that have attracted considerable attention. Unlike linear ncRNAs, such as miRNAs and lncRNAs, circRNAs are characterized by a covalently closed loop structure derived from back-splicing of precursor mRNAs. Due to their unique circularized structure, circRNAs are highly stable and are not susceptible to RNA exonuclease cleavage; thus, they have longer half-lives than other ncRNAs [51]. The biological functions of circRNAs in host cells remain mostly unexplored. To date, the most often proposed circRNA functions are miRNA sponges, which can offset miRNA-mediated mRNA inhibition. Alternative functions include transcriptional regulators, combining binding with RNA-binding proteins, and even translation to produce polypeptides [52, 53]. circRNAs can also be secreted into the intercellular space and transported into adjacent cells or body fluids to regulate cell activities [54, 55]. Compared to investigations into the roles of miRNAs in osteoclastogenesis, studies focusing on circRNAs have been limited. The available data show that circRNAs also participate in osteoclast regulation and suggest that circRNAs could function as miRNAs [2, 56].

The first study addressing the functions of circRNAs in osteoclastogenesis evaluated differences among groups of RAW264.7 cells stimulated with RANKL at four different stages. In total, 1797 circRNAs were detected in that study, and the whole expression profiles are presented. In preosteoclasts, 147 circRNAs were upregulated, and 109 circRNAs were downregulated. In mature osteoclasts, 78 circRNAs were upregulated, and 135 circRNAs were downregulated. In activated osteoclasts, 111 circRNAs were upregulated, and 45 circRNAs were downregulated. Venn diagram analysis revealed that 19 circRNAs were upregulated and 5 circRNAs were downregulated at all stages during osteoclastogenesis [2]. Although no functional characterization was performed to test the requirements for these circRNAs in osteoclastogenesis, this study triggered further research interest regarding circRNA-mediated regulation in osteoclasts.

Another study generated a microarray data set of circRNA expression during osteoclastogenesis in BMMs stimulated with RANKL and CSF1 [56]. A total of 5449 circRNAs were upregulated after induction, while 6259 were downregulated. The expression of a total of 81 circRNAs differed between groups: 29 circRNAs were upregulated, while 52 were downregulated. Among the significantly upregulated circRNAs, this study investigated the role of circRNA-28313 in BMM osteoclast differentiation. The results indicated that circRNA-28313 knockdown suppressed M-CSF and RANKL-induced differentiation of osteoclasts within BMM cells and partly prevented ovariectomy (OVX)-induced bone loss. The results of RIP assays indicated that miR-195a directly targeted circRNA-28313 and the CSF1-3′-UTR. circRNA-28313 was found to relieve miR-195a-mediated suppression of CSF1 by acting as a competing endogenous RNA (ceRNA), thereby modulating BMM osteoclast differentiation. Thus, the circRNA-28313/miR-195a/CSF1 axis can modulate osteoclast differentiation to affect OVX-induced bone absorption in mice. An in-depth analysis of the ncRNA network involved in osteoclast differentiation could enhance the understanding of the pathogenesis and treatment of abnormal bone resorption-related disease.

### Roles of extracellular vesicles in ncRNA transportation in osteoclastogenesis

Extracellular vesicles (EVs) are attracting considerable interest as intercellular communicators in many biological processes and diseases. EVs are secreted membrane vesicles that form heterogeneous populations. At present, the EVs in the major populations are usually classified into three categories according to their size, exosomes, microvesicles, and apoptotic bodies [57].

Accumulating evidence suggests that ncRNAs exhibit extracellular and paracrine functions in many physiological and pathological conditions. The roles of miRNAs have been more extensively studied than those of other ncRNAs. One of the fundamental mechanisms by which ncRNAs exit cells involves selective packaging into EVs and trafficking in the extracellular space and biofluids [58]. When EVs containing ncRNAs interact with recipient cells, they deliver their cargoes into the cytosol and thus modulate the target cells. Therefore, ncRNAs in EVs can be taken up by neighboring or distant cells and modulate those recipient cells.

#### Roles of exosomal ncRNAs in osteoclastogenesis

Exosomes are involved in osteoclastogenesis and bone resorption as intercellular communicators between bone cells. Osteoblast-derived EVs are vital tools for the delivery of active molecules such as RANKL and miRNAs to monocytes or osteoclasts. For example, RANKL-containing exosomes derived from osteoblasts activate osteoclastogenesis by activating the RANKL-RANK signaling pathway [59]. However, exosomes containing miR-503-3p inhibit osteoclastogenesis by inactivating the same pathway. In addition, exosomes derived from monocytes promote osteoclast differentiation. The final result is that osteoclasts are rapidly recruited during this phase, even though osteoclastogenesis-inhibiting exosomes are released continuously.

Newly formed osteoclasts release RANK-enriched exosomes that directly fuse with osteoblasts or competitively bind to RANKL in the extracellular matrix to regulate osteoclast formation. Osteoclast-derived exosomes can also act as inhibitors of osteogenesis. For example, exosomal miR-214-3p can inhibit osteoblast activity by targeting the 3′-UTR of cyclic AMP-dependent transcription factor (ATF4) mRNA. In vitro, exosomal miR-214-3p is transferred from osteoclasts to osteoblasts, thus triggering bone mass reduction in model mice [3].

#### Exosomes as ncRNA carriers for the balance of subchondral bone remodeling

Accumulating evidence has demonstrated a dual function of ncRNAs in osteoclast differentiation. However, unprotected ncRNAs, such as miRNAs, are easily degraded by endogenous nucleases, especially in the context of inflammatory diseases such as OA [60]. Practical and safe delivery strategies urgently need to be developed before ncRNA-mediated osteoclast targeting can be clinically translated for subchondral bone regulation in OA treatment.

**Fig. 2:**
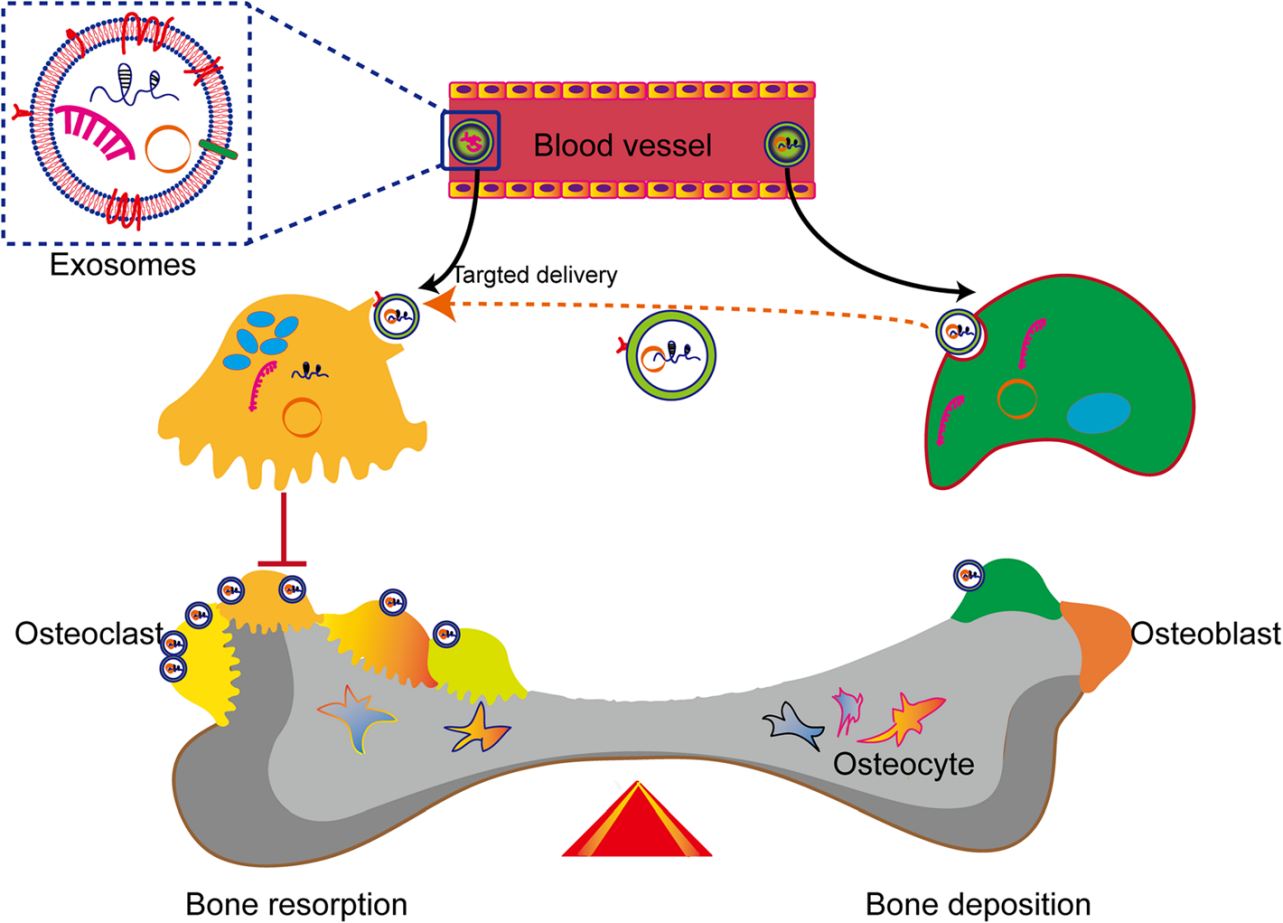
Harnessing the power of targeted exosomal circRNA/miRNA delivery for subchondral bone remodeling. These bioengineered or native exosomes could bypass biological barriers and deliver functional noncoding RNAs to recipient cells

Compared to the currently widely used delivery vectors, such as viral vectors and liposomes, exosomes have emerged as novel intracellular biomolecule carriers. Natural exosomes show better biocompatibility with higher loading ability and can bypass biological barriers than other molecular cargo carriers. Therefore, they have attracted considerable interest in the research field of nucleic drug delivery for the treatment of cancers and neurological diseases. Among delivered molecules, miRNAs are the most extensively studied ncRNAs. Additionally, circRNA delivery by exosomes has been reported [3]. Li et al. demonstrated that osteoclasts release exosomes containing miR-214-3p and could be transferred to osteoblasts to inhibit osteoblast activity and bone formation. Besides, osteoclast-targeted-delivery of antagomir-214-3p via D-Asp8-liposome encapsulation has been shown to significantly inhibit osteoclast differentiation, thus enhancing osteoblastic bone formation [3]. Given these findings, targeted exosomal delivery of ncRNAs could be used to maintain the balance of subchondral bone remodeling (Fig. 2).

## Conclusions

Osteoclast dysfunction is the critical factor that causes abnormal subchondral bone remodeling, an initiator of OA progression. In the context of osteoclastogenesis during bone resorption, miRNAs, lncRNAs, and circRNAs form an intricate epigenetic network that functions during bone resorption. However, the roles of ncRNAs in osteoclast function await further investigation both in vitro and in vivo. In-depth studies will enhance the understanding of osteoclast-initiated aberrant subchondral bone remodeling and facilitate the identification of OA treatment targets. Notably, exosomes are involved in the communication of ncRNAs, and further methods for targeting osteoclasts in subchondral bone via exosome-based ncRNA delivery should be developed.

## Data Availability

Not applicable.

## Abbreviations

ATF4: Cyclic AMP-dependent transcription factor
ATP6V0D2: ATPase H+ transporting V0 subunit D2
Bmf: BCL-2-modifying factor
BMMs: Bone marrow-derived monocytes/macrophages
ceRNA: Competing endogenous RNA
circRNAs: Circular RNAs
CSF1R: Colony-stimulating factor 1 receptor
CTSK: Cathepsin K
DC-STAMP: Dendritic cell-specific transmembrane protein
EVs: Extracellular vesicles
FOXO1: Forkhead Box O1
GSK3β: Glycogen synthase kinase-3β
IL: Interleukin
IGF2: Insulin-like growth factor 2
ncRNAs: Noncoding RNAs
LGR4: Leucine-rich repeat-containing G-protein-coupled receptor 4
lncRNAs: Long ncRNAs
MAPK: Mitogen-activated protein kinase
M-CSF: Macrophage colony-stimulating factor
MMPs: Matrix metalloproteinases
miRNAs: MicroRNAs
mRNA: Messenger RNA
NFATc1: Nuclear factor of activated T cell cytoplasmic 1
NF-κB: Nuclear actor kappa light chain enhancer of activated B cells
ncRNAs: Noncoding RNAs
OA: Osteoarthritis
OPG: Osteoprotegerin
OVX: Ovariectomy
PI3K: Phosphatidylinositol 3′-kinase
PDL: Periodontal ligament
piRNAs: Piwi-interacting RNAs
PTEN: Phosphatase and tensin homolog
RANK: Receptor activator of nuclear factor kappa-B
RANKL: Receptor activator of nuclear factor kappa-B ligand
RhoA: Ras homologous A
RIP: RNA immunoprecipitation
si-DANCR: DANCR-specific siRNA
siRNAs: Short interfering RNAs
TRAP: Tartrate-resistant acid phosphatase
Tnfsf: Tumor necrosis factor ligand superfamily member
UTRs: Untranslated regions
